# Differences in Patient Outcomes of Prevalence, Interval, and Screen-Detected Lung Cancers in the CT Arm of the National Lung Screening Trial

**DOI:** 10.1371/journal.pone.0159880

**Published:** 2016-08-10

**Authors:** Matthew B. Schabath, Pierre P. Massion, Zachary J. Thompson, Steven A. Eschrich, Yoganand Balagurunathan, Dmitry Goldof, Denise R. Aberle, Robert J. Gillies

**Affiliations:** 1 Department of Cancer Epidemiology, H. Lee Moffitt Cancer Center and Research Institute, Tampa, Florida, United States of America; 2 Thoracic Program, Vanderbilt-Ingram Cancer Center, Nashville, Tennessee, United States of America; 3 Department of Biostatistics, H. Lee Moffitt Cancer Center and Research Institute, Tampa, Florida, United States of America; 4 Department of Biomedical Informatics, H. Lee Moffitt Cancer Center and Research Institute, Tampa, Florida, United States of America; 5 Department of Cancer Imaging, H. Lee Moffitt Cancer Center and Research Institute, Tampa, Florida, United States of America; 6 Department of Computer Science and Engineering; University of South Florida, Tampa, Florida, United States of America; 7 Department of Radiological Sciences; David Geffen School of Medicine at UCLA, Los Angeles, California, United States of America; Roswell Park Cancer Institute, UNITED STATES

## Abstract

Lung cancer screening identifies cancers with heterogeneous behaviors. Some lung cancers will be identified among patients who had prior negative CT screens and upon follow-up scans develop a *de novo* nodule that was determined to be cancerous. Other lung cancers will be identified among patients who had one or more prior stable positive scans that were not determined to be lung cancer (indeterminate pulmonary nodules), but in follow-up scans was diagnosed with an incidence lung cancer. Using data from the CT arm of the National Lung Screening Trial, this analysis investigated differences in patient characteristics and survival endpoints between prevalence-, interval-, and screen-detected lung cancers, characterized based on sequence of screening results. Lung cancers immediately following a positive baseline (T0), and prior to the T1 screen, formed the prevalence cohort. Interval cancers were diagnosed following a negative screen at any time point prior to the next screening round. Two cohorts of screen-detected lung cancers (SDLC) were identified that had a baseline positive screen that was that was not determined to be lung cancer (i.e., an indeterminate pulmonary nodule), but in follow-up scans was diagnosed with an incidence lung cancer 12 (SDLC1) or 24 (SDLC2) months later. Two other incidence cohorts had screen-detected lung cancers that had baseline negative screen and upon follow-up scans developed a *de novo* nodule determined to be cancerous at 12 (SDLC3) or 24 (SDLC4) months later. Differences in patient characteristics, progression-free survival (PFS), and overall survival (OS) were assessed. The lung cancer-specific death rate was higher for SDLC3/SDLC4 compared to SDLC1/SDLC2 lung cancers (136.6/1,000 person-years vs. 71.3/1,000 person-years, P < 0.001). Moreover, PFS and OS were significantly lower for SDLC3/SDLC4 compared to SDLC1/SDLC2 (P < 0.004; P < 0.002, respectively). The findings were consistent when stratified by stage and histology. Multivariable Cox proportional models revealed that the SDLC3/SDLC4 case groups were associated with significantly poorer PFS (HR = 1.89; 95% CI 1.31–2.74) and OS (HR = 1.80; 95% CI 1.21–2.67) compared to SDLC1/SDLC2 lung cancers (HR = 1.00). Lung cancer patients who develop a *de novo* nodule that determined to be cancerous (i.e., at least one negative CT screen prior to cancer diagnosis) had poorer survival outcomes compared to patients who had at least one positive screen prior to cancer diagnosis. As such, the observation that *de novo* screen-detected are associated with poorer survival could be attributed to faster growing, more aggressive cancers that arose from a lung environment previously lacking focal abnormalities.

## Introduction

The National Lung Screening Trial (NLST) compared low-dose helical computed tomography (CT) and standard chest radiography (CXR) for three annual screens, which included a baseline prevalence screen (T0) and two annual follow-up screens (T1 and T2) [1–3]. After a median follow-up of 6.4 years, a 20% relative reduction in lung cancer mortality was observed for CT compared to CXR. Screen-detected lung cancers, defined as incidence lung cancers diagnosed in the follow-up screens at T1 and T2, accounted for 58% of all CT-detected lung cancers, were 2.7-fold higher in the CT arm, associated with a stage shift from advanced to more early stage lung cancers, and demonstrated improved 5-year survival compared to interval cancers and lung cancers diagnosed at the prevalence screen [1]. Screen-detected cancers include both a higher percentage of early stage tumors [1, 4–8] and a greater proportion of more rapidly growing cancers [4]. Additionally, screening detects indolent neoplasms that may not otherwise cause clinical symptoms or death [9]. Overdiagnosis is a potential harm of screening because the work-up and treatment of these cancers incur additional costs, patient anxiety, and morbidity for disease that may pose no mortality threat if not otherwise treated [9, 10].

Lung cancer screening with subsequent follow-up will identify screen-detected lung cancers with heterogeneous behaviors. For example, lung cancers will be diagnosed among participants who had prior negative CT screens and upon follow-up scans develop a *de novo* nodule that was determined to be cancerous. Other lung cancers will be identified among patients who had one or more prior stable positive scans that were not evaluated as lung cancer (indeterminate pulmonary nodules), but in follow-up scans was diagnosed with an incidence lung cancer. Additionally, there are screening participants that have numerous stable positive scans that are never diagnosed as lung cancer (false positives). In addition to screen-detected lung cancers, lung cancer screening also identifies prevalence cancers at the baseline screen (i.e., first screen) and interval lung cancers diagnosed following a negative screen at any time point prior to the next screening round. To date, there has not been a comprehensive analysis comparing screen-detected, prevalence, and interval lung cancers in the NLST. Additionally, it is unknown whether differences in behavior of CT screen-detected lung cancers exist based on the sequence of prior screening results, i.e., baseline positive screens vs baseline negative screens. We hypothesize that *de novo* screen-detected lung cancers that develop following prior negative CT screens are faster growing, more aggressive, and associated with poorer survival than cancers diagnosed that had prior positive screens, since the tumor arose from a lung environment previously lacking focal abnormalities. Thus, using data from the NLST data, the goal of this analysis was to compare clinical characteristics and survival outcomes of prevalence lung cancers, interval lung cancers, and screen-detected lung cancers based on the sequence of screening results.

## Methods

### NLST study population

This research was approved by the Chesapeake Institutional Review Board (Columbia, MD). De-identified NLST were obtained through the National Cancer Institute (NCI) Cancer Data Access System (CDAS) [11]. We did not generate any unique variables. As such, the same data used in our analyses are available to all researchers by submitting an application at CDAS. The NLST study design [3] and main findings [1] have been described previously. Briefly, the NLST was a randomized multicenter trial comparing screening with low-dose helical CT to CXR in high-risk individuals. Prior to randomization, participants provided signed a consent form that explained the NLST in detail, including risks and benefits. Eligibility criteria included current or former smokers aged 55 to 74 years with a minimum 30 pack-year smoking history; former smokers had to have quit within the past 15 years. The NLST staff collected data on cancer diagnoses, lung cancer progression, and deaths that occurred through December 31, 2009. We analyzed patient characteristics and survival outcomes among participants diagnosed with lung cancer in the three screenings of the CT arm (T0, T1, and T2).

### NLST CT screening results

The NLST protocol defined a positive screening result as one or more non-calcified nodules or masses measuring ≥ 4 mm in axial diameter or, less commonly, other abnormalities such as adenopathy or pleural effusion [1, 3]. Positive screens were defined in the setting of abnormalities on baseline screens or abnormalities on follow-up screens that were new, stable, or that evolved, the latter demonstrated by an increase in nodule size, consistency, or other characteristic potentially related to lung cancer. Participants with positive screening results received follow-up recommendations; trial-wide guidelines for the management of positive screens were developed, but were not mandated by protocol. The methods for classifiying and reporting identified positive lung nodules in the NLST have been described in detail elsewhere [1, 3].

Negative screens were defined as CT scans with no abnormalities, minor abnormalities not suspicious for lung cancer, or significant abnormalities not suspicious for lung cancer. Stable abnormalities across all three rounds could be classified as negative screens at the final screen (T2) at the discretion of the interpreting radiologist [1].

### Lung cancer progression and vital status

NLST staff collected data on whether cancers progressed after initial treatment [1, 3]. Progression was defined as enlargement of the original tumor, new metastasis to lymph nodes or other organ site not included in the original tumor staging, or disease recurrence. NLST staff collected vital status data on all participants; deaths were identified through update questionnaires, communication from relatives, and queries to the National Death Index. All information on the death certificate was recorded; the underlying cause of death based on the death certificate was derived according to rules established by the National Center for Health Statistics. For deaths that may have been caused by lung cancer, an Endpoint Verification Committee (EVC) reviewed medical records blinded to death certificates to determine whether the cause was lung cancer. The EVC cause of death was used in statistical analyses of the primary endpoint [1, 3].

### Prevalence-, interval-, and screen-detected lung cancers

We restructured the entire CT arm of the NLST (S1 Fig) according to baseline and follow-up screening results (positive vs. negative screen). Lung cancers immediately following a positive baseline (T0), and prior to the T1 screen, formed the prevalence cases (i.e., baseline lung cancers). The screen-detected lung cancers were defined based on specific sequences of screening results and formed six screen-detected lung cancer case groups. Screen-Detected Lung Cancer Cohorts 1, 2, and 5 had a baseline positive screen that was that was determined to be lung cancer (i.e., an indeterminate pulmonary nodule), but in follow-up scans was diagnosed with an incidence lung cancer 12 (T1) or 24 (T2) months later. Screen-Detected Lung Cancer Cohorts 3, 4, and 6 has a baseline negative screen and upon follow-up scans develop a *de novo* nodule that determined to be cancerous either 12 (T1) or 24 (T2) months later.

- Screen-Detected Lung Cancer Cohort 1 (SDLC1) (T0 positive→T1 positive→lung cancer diagnosis) had baseline positive screens not associated with a lung cancer diagnosis, and then a screen-detected lung cancer followed a positive screen at T1.
- Screen-Detected Lung Cancer Cohort 2 (SDLC2) (T0 positive→T1 positive→T2 positive→lung cancer diagnosis) had baseline and T1 positive screens not associated with a lung cancer diagnosis, and then a screen-detected lung cancer followed a positive screen at T2.
- Screen-Detected Lung Cancer Cohort 3 (SDLC3) (T0 negative→T1 positive→lung cancer diagnosis) had baseline negative screens, and then a screen-detected lung cancer followed a positive screen at T1
- Screen-Detected Lung Cancer Cohort 4 (SDLC4) (T0 negative→T1 negative→T2 positive→lung cancer diagnosis) had baseline and T1 negative screens, and then a screen-detected lung cancer followed a positive screen at T2.
- Screen-Detected Lung Cancer Cohort 5 (SDLC5) (T0 positive→T1 negative→ T2 positive→lung cancer diagnosis) had baseline positive screens not associated with a lung cancer diagnosis, and negative screen at T1, and then a screen-detected lung cancer followed a positive screen at T2.
- Screen-Detected Lung Cancer Cohort 6 (SDLC6) (T0 negative→T1 positive→T2 positive→lung cancer diagnosis) had a baseline negative screen, a T1 positive screen not associated with a lung cancer diagnosis, and a screen-detected lung cancer followed a positive screen at T2.

‘Interval cancers’ are defined as cancers that occur between scheduled screening episodes and the previous screening episode was negative or “normal”. In the NLST, interval cancers were defined as lung cancers diagnosed following a negative screen at any time point prior to the next screening round.

- All eighteen T0 interval lung cancers had a baseline negative screen.
- All ten T1 interval lung cancers had baseline and T1 negative screens.
- Among the sixteen T2 interval lung cancers, ten had negative screens at T0, T1, and T2; two had negative screens at T0, positive screens at T1, and then negative screens at T2; two had positive screens at T0, and then negative screens at T1 and T2; and two had positive screens at T0 and T1, and then negative screens at T2.

### Statistical analyses

Pearson’s chi-square was used to test for differences in categorical patient characteristics across the case cohorts and ANOVA, Van Der Waerden normal scores test, and Wilcoxon rank-sum test were used test for differences in continuous variables. Survival analyses were performed using Kaplan-Meier survival curves, the log-rank statistic, and multivariable Cox proportional hazard models. Progression-free survival (PFS) and overall survival (OS) were assessed from date of lung cancer diagnosis to the date of an event or date of last follow-up. For PFS an event was defined as death or progression of cancer; for OS an event was defined as death. Among individuals without an event, censoring occurred at either 5-years or date of last follow-up if less than 5-years. Overall and lung cancer-specific death rates per 1,000 person-years were also calculated. All statistical analyses were two-sided and performed using SAS version 9.3 and Stata/MP 12.1 for Windows (32-bit).

## Results

### Screening results and screening intervals

**Fig 1:**
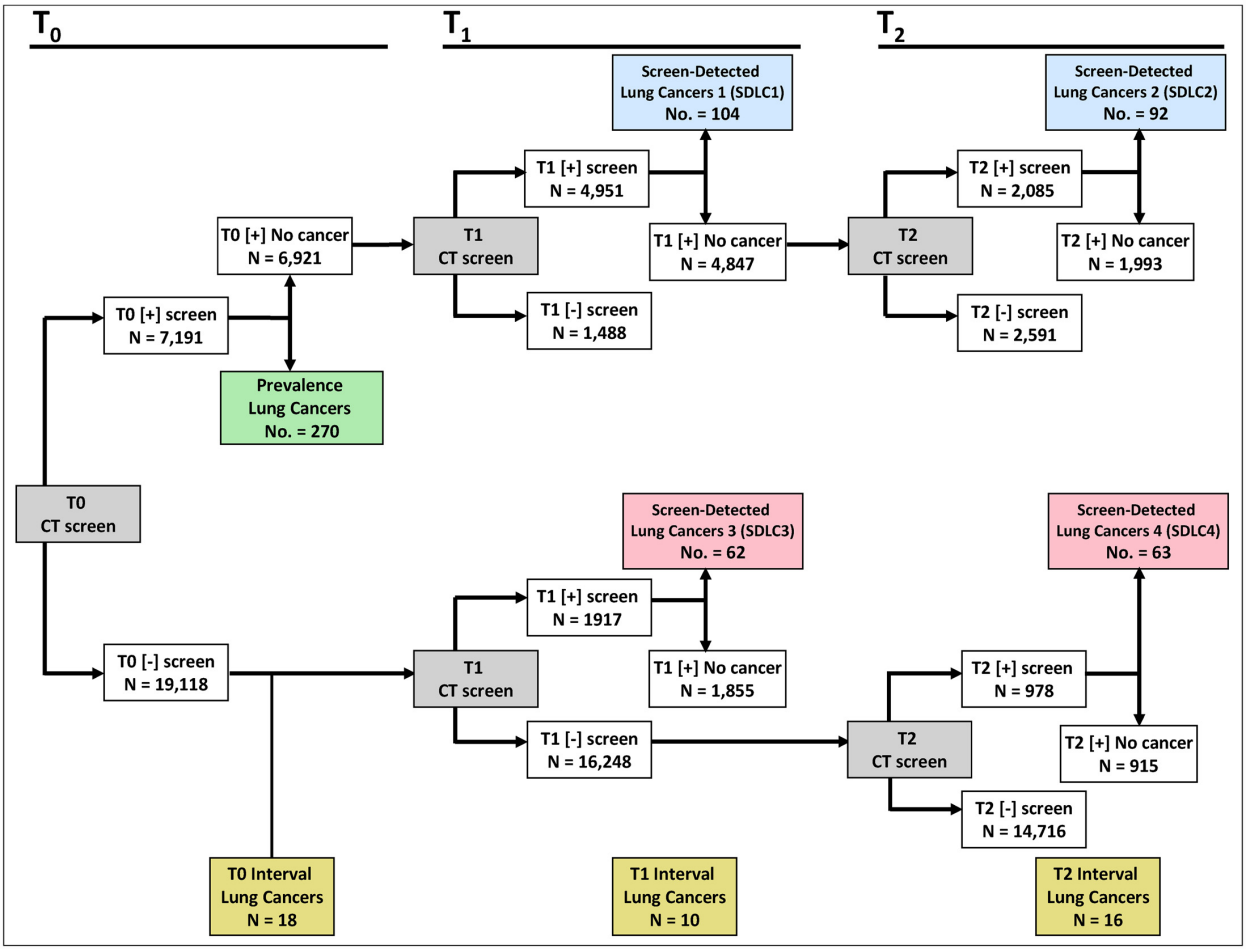
Schema for the lung cancer case cohorts in the NLST at prevalence (baseline) and incidence screening (follow-up) rounds. Abbreviations: T0 = baseline screen; T1 = first screen; T2 = second screen; [+] = positive screen; [–] = negative screen. Prevalence lung cancers diagnosed at T0 are shaded green. Screen-detected lung cancers diagnosed at T1 and T2 rounds in which T0 screens were positive are shaded blue. Screen-detected lung cancers diagnosed at T1 and T2 in which T0 screens were negative are shaded red. Interval cancers (beige boxes) were diagnosed following a negative screen. Participants were excluded if their screening results were inadequate or not compliant. At baseline we excluded 413 screens, 1,471 screens at T1, and 713 screens at T2.

The schema presented in Fig 1 represents the cohorts that were investigated in this analysis. We excluded SDLC5 and SDLC6 (S1 Fig) since these patients cannot be collapsed into the other case groups because their sequence of screening results are incompatible with SDLC1 and SDLC2 and with SDLC3 to SDLC4. Moreover, they were not analyzed as independent case groups because of their small sample sizes. Our sample size slightly varies from previous analyses [12] since 78 NLST participants who were deemed ineligible (e.g., quit smoking > 15 years) after enrollment and randomization were still included in our analyses. Despite their ineligible status, these 78 participants remained in the CT-arm of the trial and only 5 of the 78 participants were diagnosed with lung cancer (3 prevalence, 1 SDLC1, and 1 interval).

There were 270 prevalence cancers, 44 interval cancers, and SDLC1 and SDLC2 case groups included 104 and 92 lung cancers, respectively. For the SDLC1 cases, 66.4% of the lung cancers were observed in positive screens in which new or evolving nodules (increase in size or consistency) were observed (S1 Table). Similarly, 87% of SDLC2 lung cancers were observed in the setting of new or evolving nodules. Similarly, for the SDLC3 and SDLC4 case groups, 85.5% and 98.4% of lung cancers were associated with positive screens in which new or evolving nodules were observed, respectively. There were no significant differences between the T1 screen-detected incidence cancers (SDLC1 and SDLC3) or the T2 screen-detected incidence cancers (SDLC2 and SDLC4) with respect to time from randomization to diagnosis or time interval between successive screening visits (S2 Table).

### Demographic and clinical differences between prevalence, screen-detected, and interval lung cancers

There were no significant differences between SDLC1 versus SDLC2 and between SDLC3 versus SDLC4 for patient characteristics (Table 1) and for PFS and OS (S2 Fig). Thus, for all subsequent analyses we combined SDLC1 and SDLC2 into one group representing incidence lung cancers that had baseline positive screens SDLC3 and SDLC4 into one group representing incidence lung cancers that had baseline negative screens. In Tables 2 and 3, pairwise comparisons were assessed for the demographic and clinical characteristics between combined SDLC1/2 cases and SDLC3/4 cases, prevalence cases, and interval cases.

**Table 1:** Baseline Demographics and Clinical Characteristics of Four Screen-Detected Lung Cancer Case Cohorts.

Characteristic^5^	SDLC1	SDLC2	P-value^1^	SDLC3	SDLC4	P-value^2^
	(N = 104)	(N = 92)		(N = 62)	(N = 63)	
**Age, mean (SD)**	64.4 (5.2)	63.0 (4.8)	0.056	63.4 (4.9)	63.8 (5.6)	0.667
**Sex, N (%)**						
Male	59 (56.7)	51 (55.4)	0.412	39 (62.9)	44 (69.8)	0.855
Female	45 (43.3)	41 (44.6)		23 (37.1)	19 (30.2)	
**Race, N (%)**						
White	98 (94.2)	87 (94.8)	0.221	56 (90.3)	57 (90.5)	0.630
Black	5 (4.8)	5 (5.4)		4 (6.5)	0 (0.0)	
Asian	1 (1.0)	0 (0.0)		1 (1.6)	3 (4.8)	
Other	0 (0.0)	0 (0.0)		1 (1.6)	3 (4.8)	
**Ethnicity, N (%)**						
Hispanic or Latino	0 (0.0)	0 (0.0)	0.312	1 (1.6)	0 (0.0)	0.286
Neither Hispanic/Latino	104 (100.0)	91 (98.91)		61 (98.4)	63 (100.0)	
Unknown	0 (0.0)	1 (1.09)		0 (0.0)	0 (0.0)	
**Smoking, N (%)**						
Current	53 (51.0)	52 (56.5)	0.115	42 (67.7)	34 (54.0)	0.436
Former	51 (49.0)	40 (43.5)		20 (32.3)	29 (46.0)	
**Pack-Years, mean (SD)**						
Current smokers	62.2 (28.7)	62.3 (20.1)	0.985	69.2 (25.7)	64.5 (21.8)	0.401
Former smokers	68.2 (27.2)	62.6 (28.2)	0.340	66.8 (25.5)	64.6 (27.0)	0.773
**Education, N (%)**						
8th grade or less	1 (1.0)	1 (1.1)	0.694	1 (1.6)	1 (1.6)	0.753
11th grade	6 (5.8)	4 (4.4)		1 (1.6)	4 (6.4)	
High school graduate/GED	31 (29.8)	22 (23.9)		16 (25.8)	12 (19.1)	
Post high school training, excluding college	19 (18.3)	17 (18.5)		12 (19.4)	7 (11.11)	
Associate degree/some college	22 (21.2)	16 (17.4)		17 (27.4)	20 (31.75)	
Bachelor’s Degree	11 (10.6)	15 (16.3)		8 (12.9)	12 (19.05)	
Graduate School	12 (11.5)	14 (15.2)		6 (9.9)	6 (9.52)	
Other	1 (1.0)	3 (3.3)		1 (1.6)	1 (1.59)	
Unknown	1 (1.0)	0 (0.0)		1 (1.6)	1 (1.59)	
**Marital, N (%)**						
Never married	4 (3.9)	5 (5.4)	0.394	1 (1.61)	3 (4.8)	0.558
Married	69 (66.4)	54 (58.7)		39 (62.9)	44 (69.8)	
Widowed	9 (8.7)	7 (7.6)		5 (8.1)	7 (11.1)	
Separated	0 (0.0)	0 (0.0)		2 (3.2)	1 (1.6)	
Divorced	21 (20.2)	26 (28.3)		15 (24.2)	8 (12.7)	
Unknown	1 (1.0)	0 (0.0)		1 (1.6)	3 (4.8)	
**History of COPD, N (%)**						
Yes	8 (7.7)	8 (8.7)	0.973	8 (12.9)	8 (12.7)	0.798
No	96 (92.3)	84 (91.3)		54 (87.1)	55 (87.3)	
**FH of lung cancer, N (%)**						
Yes	26 (25.0)	21 (22.8)	0.796	16 (25.8)	15 (23.8)	0.722
No	78 (75.0)	71 (77.2)		46 (74.2)	48 (76.2)	
**Stage, N (%)**^3^						
IA	58 (55.8)	45 (48.9)	0.922	28 (45.2)	37 (58.7)	0.626
IB	13 (12.5)	16 (17.4)		5 (8.1)	4 (6.4)	
IIA	5 (4.8)	2 (2.2)		6 (9.7)	5 (7.9)	
IIB	4 (3.8)	2 (2.2)		2 (3.2)	1 (1.6)	
IIIA	6 (5.8)	8 (8.7)		6 (9.7)	4 (6.4)	
IIIB	8 (7.7)	6 (6.5)		5 (8.1)	4 (6.4)	
IV	9 (8.7)	12 (13.0)		8 (12.9)	6 (9.5)	
Carcinoid, not assessed	0 (0.0)	1 (1.09)		0 (0.0)	0 (0.0)	
Unknown	1 (1.0)	0 (0.0)		2 (3.23)	2 (3.17)	
**Tumor Size, N, mean mm (SD)**	97, 20.5 (15.7)	85, 22.5 (17.1)	0.496	56, 24.1 (23.3)	60, 20.6 (15.4)	0.340
**Histology, N (%)**						
Adenocarcinoma^4^	67 (64.4)	50 (54.6)	0.280	21 (33.9)	20 (32.8)	0.244
Small cell carcinoma	4 (3.9)	9 (9.8)		11 (17.7)	6 (9.5)	
Squamous cell carcinoma	18 (17.3)	15 (16.3)		16 (25.8)	24 (38.1)	
Other and NOS	15 (14.4)	17 (18.5)		14 (22.6)	11 (17.5)	
Unknown	0 (0.0)	1 (1.1)		0 (0.0)	2 (3.2)	
**Cause of death, N (%)**						
Death due to lung cancer	29 (27.9)	24 (26.1)		31 (50.0)	23 (36.5)	
Death not due to lung cancer	8 (7.7)	3 (3.3)	0.359	4 (6.5)	4 (6.4)	0.285
Cause of death unknown	0 (0.0)	0 (0.0)		1 (1.6)	0 (0.0)	
No death reported	67 (64.4)	65 (70.7)		26 (41.9)	36 (57.1)	
**5-year Survival Rate, %**	65.9%	67.5%	0.893	41.0%	55.2%	0.114
**Death rate per 1,000 person-years, (95% CI)**						
Overall	83.3	90.4	0.119	183.4	135.6	0.372
	(58.7–112.2)	(62.0–131.8)		(128.5–248.0)	(89.4–191.4)	
Lung cancer-specific	65.3	80.3	0.227	157.9	115.5	0.129
	(45.4–94.0)	(53.9–119.9)		(107.3–218.2)	(73.2–167.3)	

Abbreviations: SDLC = Screen-Detected Lung Cancers; GED = graduate education degree; COPD = chronic obstructive pulmonary disease; FH = Family history; Pack-years = packs smoked/day x years smoked;

^1^ P-value comparing SDLC1 vs. SDLC2

^2^ P-value comparing SDLC3 vs. SDLC4

^3^ Carcinoid and unknown were removed before testing with Pearson’s chi-square

^4^ BAC and adenocarcinoma were combined into one group.

^5^ Demographic data were self-reported.

**Table 2:** Baseline Demographic of the Grouped Screen-Detected, Prevalence, and Interval Cancer Cohorts.

Characteristic^5^	SDLC1/SDLC2	SDLC3/SDLC4	P-Value^1^	Prevalence	P-Value^2^	Interval	P-Value^3^
	(N = 196)	(N = 125)		(N = 270)		(N = 44)	
**Age, mean years (SD)**	63.7 (5.1)	63.6 (5.3)	0.767	63.0 (5.1)	0.897	63.0 (5.1)	0.799
**Sex**			0.067		0.167		**0.049**
Male, N (%)	110 (56.1)	83 (66.4)		157 (58.2)		33 (75.00)	
Female, N (%)	86 (43.9)	42 (33.6)		113 (41.9)		11 (25.00)	
**Race, N (%)**^4^			0.177		0.263		0.577
Whites	185 (94.4)	113 (90.4)		250 (92.9)		40 (90.91)	
Other	11 (5.6)	12 (9.6)		20 (7.4)		4 (9.09)	
**Smoking Status, N (%)**			0.203		0.427		0.334
Current	105 (53.6)	76 (60.8)		149 (55.2)		29 (65.91)	
Former	91 (46.4)	49 (39.2)		121 (44.8)		15 (34.09)	
**Pack-years Smoked, Mean (SD)**							
Current smokers	62.2 (24.7)	67.1 (24.0)	0.186	65.5 (29.3)	0.444	67.3 (26.2)	0.506
Former smokers	65.7 (27.6)	65.5 (26.1)	0.960	65.5 (30.5)	0.999	69.4 (31.3)	0.973
**History of COPD, N (%)**			0.176		0.393		0.482
Yes	16 (18.2)	16 (12.8)		29 (10.7)		3 (6.82)	
No	180 (91.8)	109 (87.2)		241 (89.3)		41 (93.18)	
**FH of Lung Cancer, N (%)**			0.867		0.391		0.227
Yes	47 (24.0)	31 (24.8)		79 (29.3)		7 (15.91)	
No	149 (76.0)	94 (75.2)		191 (70.7)		37 (84.09)	

Abbreviations: SDLC = Screen-Detected Lung Cancers, SD = Standard deviation, Pack-years = packs smoked/day x years smoked; COPD = chronic obstructive pulmonary disease; FH = Family history in a direct relative.

Statistically significant p-values (p < 0.05) are shown in **bold.**

^1^P-value comparing SDLC1 and SDLC2 vs. SDLC3 and SDLC4

^2^ P-value comparing SDLC1/SDLC2 vs. SDLC3/SDLC4 vs. prevalence cancers

^3^ P-value comparing SDLC1/SDLC2 vs. SDLC3/SDLC4 vs. prevalence vs. interval cancers.

^4^ For SDLC1 and SDLC2 99.5% were neither Hispanic/Latino, 99.2% for SDLC3 and SDLC4, and 96.4% for prevalence cancers

^5^ Demographic data were self-reported. Level of education and marital status were not significantly different between cohorts and are not shown.

**Table 3:** Clinical Characteristics and Outcomes of the Grouped Screen-Detected, Prevalence, and Interval Cancer Cohorts.

Characteristic	SDLC1/SDLC2	SDLC3/SDLC4	P-Value^1^	Prevalence	P-Value^2^	Interval	P-Value^3^
	(N = 196)	(N = 125)		(N = 270)		(N = 44)	
**Lung Cancer Histology, N (%)**^4^			**< 0.001**		**< 0.001**		**< 0.001**
Adenocarcinoma	117 (60.0)	41 (33.8)		146 (54.1)		8 (18.2)	
Small cell carcinoma	13 (6.5)	17 (13.6)		15 (5.6)		14 (31.8)	
Squamous cell carcinoma	33 (16.8)	40 (32.0)		46 (17.1)		13 (29.6)	
Other and NOS	32 (16.3)	25 (20.0)		60 (22.3)		9 (20.5)	
Unknown	1 (0.5)	2 (1.6)		3 (1.1)		0 (0.0)	
**Stage, N (%)**^5^			0.437		0.206		**< 0.001**
Stage I	132 (67.3)	74 (59.2)		156 (57.8)		7 (15.9)	
Stage II	13 (6.6)	14 (11.2)		18 (6.7)		5 (11.4)	
Stage III	28 (14.3)	19 (15.2)		52 (19.3)		18 (40.9)	
Stage IV	21 (10.7)	14 (11.2)		41 (15.2)		14 (31.8)	
Carcinoid, not assessed	1 (0.5)	0 (0.0)		2 (0.74)		0 (0.0)	
Unknown	1 (0.5)	4 (3.2)		1 (0.37)		0 (0.0)	
**Tumor Size,mean mm (SD)**	21.3 (16.3)	22.3 (19.6)	0.623	25.5 (17.2**)**	**0.032**	41.4 (21.7)	**< 0.001**
**Cause of Death, N (%)**			**0.007**		**0.028**		**< 0.001**
Death due to lung cancer	53 (27.0)	54 (43.2)		120 (40.4)		35 (79.55)	
Death not due to lung cancer	11 (5.6)	8 (6.4)		18 (6.1)		2 (4.55)	
Cause of death unknown	0 (0.0)	1 (0.8)		1 (0.4)		0 (0.0)	
No death reported	132 (67.4)	62 (49.6)		158 (53.2)		7 (15.91)	
**5-year Survival Rate, %**	65.7	47.6	**< 0.001**	59.9	**0.004**	17.3%	**< 0.001**
**Death rate per 1,000 person-years, (95% CI)**							
**Overall**	86.2	159.6	**< 0.001**	98.9	NC	453.5	NC
	(66.3–108.5)	(122.5–201.1)		(82.0–117.4)		(319.3–610.9)	
**Lung cancer-specific**	71.3	136.6	**< 0.001**	84.1	NC	429.0	NC
	(53.4–91.8)	(102.6–175.4)		(68.6–101.2)		(298.8–582.4)	

Abbreviations: SDLC = Screen-Detected Lung Cancers; SD = Standard deviation; NC = not calculated

Statistically significant p-values (p < 0.05) are shown in **bold.**

^1^ P-value comparing SDLC1/SDLC2 vs. SDLC3/SDLC4.

^2^ P-value comparing SDLC1/SDLC2 vs. SDLC3/SDLC4 vs. prevalence cancers.

^3^ P-value comparing SDLC1/SDLC2 vs. SDLC3/SDLC4 vs. prevalence vs. interval cancers.

^4^ BAC and adenocarcinoma were combined into one group. Unknown removed before testing with Pearson’s chi-square.

^5^ Carcinoid and unknown removed before testing with Pearson’s chi-square.

There were no significant differences in demographic characteristics between the various cohorts with the exception of sex (Table 2). A higher percentage of interval cancers were observed in men (75%) than in the other cohorts (56.1% for SDLC1/2, 66.4% for SDLC3/4, and 58.2% for prevalence lung cancers; P = 0.049).

There were significant differences in histological subtypes of lung cancer between the two combined incidence cohorts (Table 3). Specifically, the SDLC1/2 cases had a higher percentage of adenocarcinoma histology than the SDLC3/4 cases (60.0% vs. 33.8%). Conversely, the SDLC3/4 cases had a significantly higher percentage of squamous cell carcinomas compared to the SDLC1/2 cases (32% vs. 16.8%; P < 0.001). The prevalence lung cancers had a similar distribution of histological subtypes compared to SDLC1/2 cases which was confirmed by pairwise comparison analysis (P = 0.503, not included in Table 3). However, the pairwise comparison between SDLC3/4 and prevalence lung cancers was statistically significant (P < 0.001, not included in Table 3). As such, the prevalence lung cancers had a significantly (P < 0.001) higher percentage of adenocarcinomas (54.1%) compared to the SDLC1/2 cases (33.8%). Interval lung cancers had the highest percentage of small cell carcinomas (31.8%) compared to SDLC1/2 (6.5%), SDLC3/4 (13.6%) and prevalence lung cancers (5.6%).

The only significant difference in stage distribution was that interval lung cancers had a significantly higher percentage of stage III (40.9%) and IV (31.8%) lung cancers (P < 0.001) compared to SDLC1/2 (14.3% and 10.7%), SDLC3/4 (15.2% and 11.2%) and prevalence lung cancers (15.2% and 19.3%). The mean tumor size was significantly higher for prevalence lung cancers (25.5 mm SD = 17.2) and interval lung cancers (41.1 mm SD = 21.7) compared to SDLC1/2 (21.3 mm SD = 16.3) and SDLC3/4 lung cancers (22.3 mm SD = 19.6).

### Patient outcomes and Kaplan-Meier survival analyses

Cause of death (Table 3) was significantly different between the combined screen-detected cases (P = 0.007). The absolute number and percentage of participants in the SDLC1/2 cases with no reported deaths were significantly higher than in the SDLC3/4 cases (132 participants [67.4%] vs. 62 participants[49.6%]). Lung cancer deaths were nearly equal in number between the two combined screen-detected cohorts, but the percentage was significantly lower in the SDLC1/2 cases (27.0% vs. 43.2%). There was also a significant difference in all cause deaths when comparing the combined screen-detected cases to prevalence cases (P = 0.028), largely driven by the reduced lung cancer death rate in the SDLC1/2 cases. Lung cancer deaths were highest in those with interval cancers (79.6%), which was significantly different from the screen-detected and prevalence cases (P < 0.001). The overall- and lung cancer-specific death rates were significantly higher for the SDLC3/4 cases (159.6 per 1000 person-years and 136.6 per 1000 person-years) compared to SDLC1/2 cases (86.2 per 1000 person-years and 71.3 per 1000 person-years).

**Fig 2:**
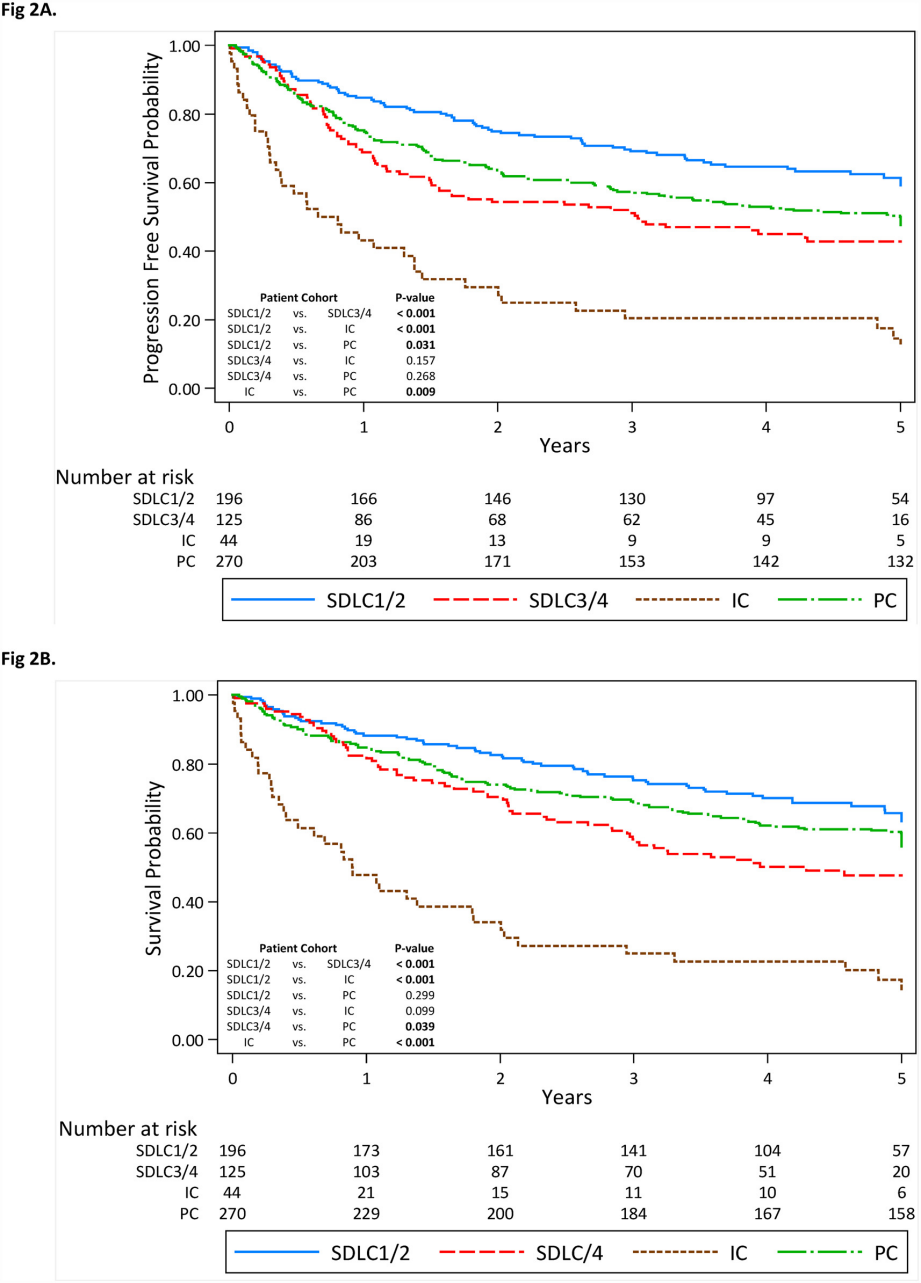
Kaplan-Meier estimates and number of subjects at risk for (A) progression free survival and (B) overall survival in the prevalence-, interval-, and grouped screen-detected lung cancers. Abbreviations: SDLC1/2 = Combined screen-detected lung cancers following positive screens at T1 and T2 rounds in cohorts with T0 positive screens; SDLC3/4 = Combined screen-detected lung cancers following positive screens at T1 and T2 rounds among cohorts with T0 or T0 and T1 negative screens; PC = Prevalence lung cancer cohort; IC = Interval cancer cohort.

Kaplan-Meier survival analyses were used to calculate 5-year survival rates (Table 3) and compare differences in PFS and OS between the lung cancer case groups. Across all lung cancer case groups, PFS (Fig 2A) was significantly highest in the SDLC1/2 case group than the SDLC3/4 cases (P < 0.001), prevalence (P = 0.031), and interval cancer cases (P < 0.001). Similar findings were observed for OS (Fig 2B). PFS and OS were lowest in the interval cancers; PFS and OS for the prevalence cancers was intermediate between the combined screen-detected cases.

**Fig 3:**
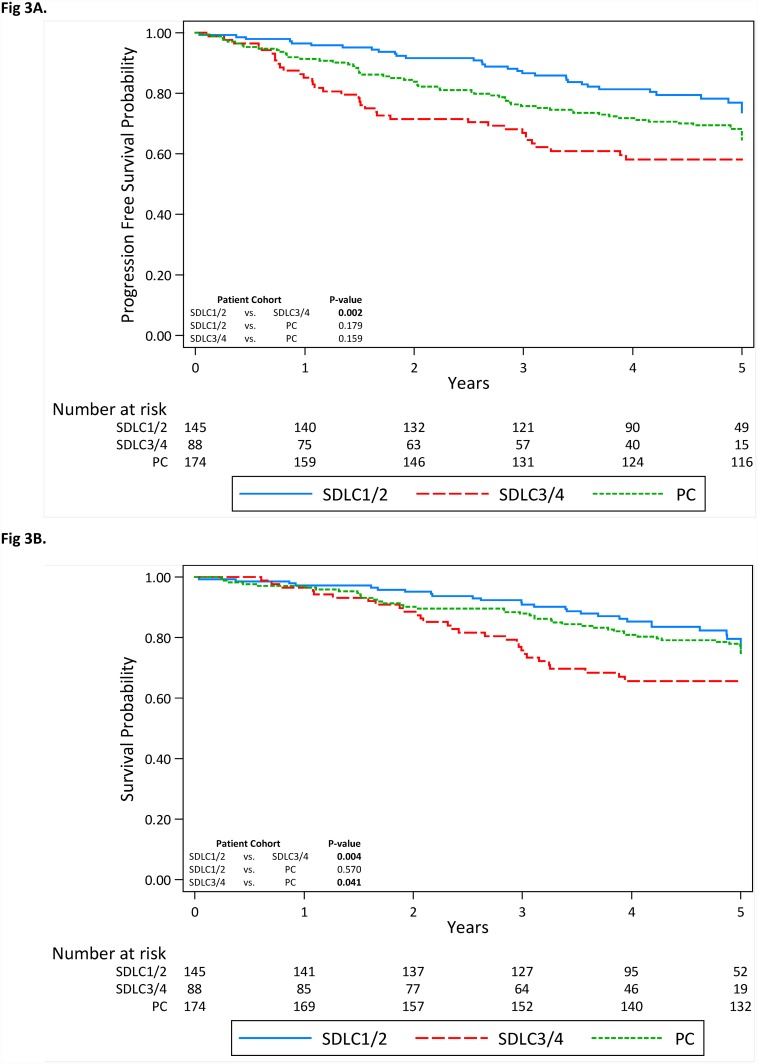
Kaplan-Meier estimates and number of subjects at risk in stage I and II patients for (A) progression free survival and (B) overall survival in the prevalence-, interval-, and grouped screen-detected lung cancers. Abbreviations: SDLC1/2 = Combined screen-detected incidence lung cancers following positive screens at T1 and T2 rounds in cohorts with T0 positive screens; SDLC3/4 = Combined screen-detected incidence lung cancers following positive screens at T1 and T2 rounds among cohorts with T0 or T0 and T1 negative screens.

When analyzing only early stage lung cancers (stages I and II), both PFS and OS remained higher in the SDLC1/2 cases compared to the SDLC3/4 cases (Fig 3A and 3B; P < 0.002, P < 0.004, respectively); differences in survival endpoints between the SDLC1/2 and prevalence lung cancers were not significant. The SDLC3/4 cases had significantly poorer OS than the prevalence cases (Fig 3B, P = 0.041). When OS and PFS were stratified by histology (S3 and S4 Figs), SDLC3/4 cases consistently had poorer outcomes than the SDLC1/2 cases.

### Multivariable hazard models for PFS and OS

Multivariable Cox proportional hazard models of PFS and OS (Table 4) were utilized to account for confounding from demographic and clinical variables that may have contributed to the survival differences observed between the screen-detected lung cancer case groups. Model variables included age, sex, race, smoking variables, self-reported history of COPD, family history of lung cancer, lung cancer stage, and histology. For the PFS analyses, we also included treatment as a covariate. The SDLC1/2 cases were set as the reference group (Hazard Ratio [HR] = 1.0), and separate analyses were performed assessing SDLC3/4 combined and for independent point estimates for SDLC3 and SDLC4. Relative to SDLC1/2, the hazard ratio for PFS was worst for the combined SDLC3/4 cases (HR = 1.89; 95% confidence interval [CI] 1.31–2.74) as well as for SDLC3 (HR = 1.75; 95% CI 1.13–2.74) and SDLC4 (HR = 2.07; 95% CI 1.31–3.25). Similarly, the models for OS revealed that the combined SDLC3/4 cases (HR = 1.80; 95% CI 1.21–2.67) as well as the SDLC3 (HR = 1.88; 95% CI 1.19–3.01) and SDLC4 (HR = 1.71; 95% CI, 1.04–2.81) cohorts were associated with significantly elevated HRs. For the PFS analyses, family history of lung cancer, stage of disease, and treatment exhibited significantly elevated HRs. For the OS analyses, age and stage of disease exhibited significantly elevated HRs.

**Table 4:** Multivariable Cox Proportional Hazards Models for Progression Free and Overall Survival for the Screen-Detected Cohorts.

Characteristic	Progression Free Survival					Overall Survival			
	HR		(95% CI)	HR	(95% CI)	HR	(95% CI)	HR	(95% CI)
**Screen-detected lung cancers**									
SDLC1/2	1.00	(Reference)		1.00	(Reference)	1.00	(Reference)	1.00	(Reference)
SDLC3/4	**1.89**	**(1.31–2.74)**		-	-	**1.80**	**(1.21–2.67)**		
SDLC3	-	-		**1.75**	**(1.13–2.74)**	-	-	**1.88**	**(1.19–3.01)**
SDLC4	-	-		**2.07**	**(1.31–3.25)**	-	-	**1.71**	**(1.04–2.81)**
**Age, per 1 year**	1.03	(0.99–1.07)		1.03	(0.99–1.07)	**1.06**	**(1.02–1.10)**	**1.06**	**(1.02–1.10)**
**Sex**									
Female	1.00	(Reference)		1.00	(Reference)	1.00	(Reference)	1.00	(Reference)
Male	0.96	(0.67–1.39)		0.97	(0.88–1.41)	1.39	(0.91–2.21)	1.39	(0.91–2.11)
**Race**									
White	1.00	(Reference)		1.00	(Reference)	1.00	(Reference)	1.00	(Reference)
Non-white	0.77	(0.39–1.52)		0.75	(0.38–1.50)	0.96	(0.47–1.98)	0.98	(0.47–2.03)
**Smoking Status**									
Former	1.00	(Reference)		1.00	(Reference)	1.00	(Reference)	1.00	(Reference)
Current	0.98	(0.69–1.39)		1.00	(0.70–1.42)	1.07	(0.73–1.57)	1.06	(0.72–1.56)
**Pack-years, per 1 pack-year**	1.00	(0.99–1.01)		1.00	(0.99–1.01)	0.99	(0.99–1.01)	1.00	(0.99–1.01)
**History of COPD**									
No	1.00	(Reference)		1.00	(Reference)	1.00	(Reference)	1.00	(Reference)
Yes	1.57	(0.89–2.78)		1.60	(0.91–2.83)	1.31	(0.71–2.43)	1.31	(0.70–2.42)
**FH of lung cancer**									
No	1.00	(Reference)		1.00	(Reference)	1.00	(Reference)	1.00	(Reference)
Yes	**1.88**	**(1.27–2.80)**		**1.86**	**(1.25–2.78)**	1.52	(0.98–2.32)	1.53	(0.99–2.37)
**Stage at Diagnosis**									
Stage IA	1.00	(Reference)		1.00	(Reference)	1.00	(Reference)	1.00	(Reference)
Stage IB	1.38	(0.69–2.72)		1.38	(0.70–2.74)	1.53	(0.71–3.31)	1.53	(0.71–3.30)
Stage IIA + IIB^1^	**4.04**	**(2.14–7.61)**		**4.07**	**(2.16–7.67)**	**4.08**	**(2.12–7.85)**	**4.08**	**(2.12–7.85)**
Stage IIIA + IIIB^1^	**6.38**	**(3.72–10.96)**		**6.42**	**(3.74–11.03)**	**8.27**	**(4.94–13.84)**	**8.22**	**(4.90–13.78)**
Stage IV	**15.22**	**(7.94–29.19)**		**15.48**	**(8.05–29.75)**	**25.94**	**(14.08–47.79)**	**25.64**	**(13.86–47.40)**
Unknown	1.30	(0.29–5.82)		1.32	(0.29–5.86)	2.27	(0.50–10.36)	2.25	(0.49–10.26)
**Histology**									
Adenocarcinoma^2^	1.00	(Reference)		1.00	(Reference)	1.00	(Reference)	1.00	(Reference)
Squamous cell carcinoma	1.19	(0.74–1.91)		1.17	(0.72–1.87)	1.59	(0.96–2.64)	1.60	(0.96–2.67)
Small cell carcinoma	1.05	(0.64–1.71)		0.75	(0.41–1.38)	1.64	(0.90–2.99)	1.65	(0.90–3.01)
**Treatment**^3^									
Surgery	1.00	(Reference)		1.00	(Reference)	--	--	--	--
Chemotherapy and other	**2.64**	**(1.43–4.85)**		**2.68**	**(1.45–4.96)**	--	--	--	--
Radiation therapy	1.67	(0.99–2.81)		**1.71**	**(1.01–2.89)**	--	--	--	--

Abbreviations: HR = hazard ratio; CI = confidence interval; SDLC = Screen-Detected Lung Cancers; BAC = bronchioloalveolar cell carcinoma; NOS = not otherwise specified; FH = family history; COPD = Chronic Obstructive Pulmonary Disease

Statistically significant hazard ratios (p < 0.05) are shown in **bold.**

^1^ Due to small sample sizes, some stages were collapsed.

^2^ BAC and adenocarcinoma were combined into one group

^3^ Treatment was only included in the PFS analyses

## Discussion

We performed a *post hoc* analysis of the CT arm of the NLST to investigate differences in patient characteristics and outcomes between prevalence lung cancers, interval lung cancers and screen-detected incidence cancers. Furthermore, we investigated whether there were differences among screen-detected incidence cancers in which only positive screens preceded the diagnosis of lung cancer (SDLC1 and SDLC2 case groups) and screen-detected incidence cancers with one or more antecedent negative screens (SDLC3 and SDLC4 case groups). Because our analyses revealed no significant differences in patient characteristics and outcomes between SDLC1 and SDLC2 and between SDLC3 and SDLC4, we collapsed the four screen-detected case groups into the two combined case groups (SDLC1/SDLC2 and SDLC3/SDLC4). Thus, the main finding of this report is that lung cancer patients who develop a *de novo* nodule that determined to be cancerous (i.e., at least one negative CT screen prior to cancer diagnosis) had poorer survival outcomes compared to patients who had at least one positive screen prior to cancer diagnosis. As such, the observation that *de novo* screen-detected are associated with poorer survival could be attributed to faster growing, more aggressive cancers that arose from a lung environment previously lacking focal abnormalities.

Previous *post hoc* analyses of CT screening studies have found survival advantages for lung cancer based on sex, smoking status, pack-year smoked, stage, and histology [13–15]. Our analysis is among the first to show differences in patient survival outcomes among screen-detected lung cancers based on the sequence of antecedent screening results (i.e., positive screen versus negative screen). We performed additional analyses to rule out potential biases that may have contributed to the observed findings. First, we found no differences in the time from randomization (S2 Table) to cancer diagnoses for the T1 screen-detected cases (SDLC1 and SDLC3) and for the T2 screen-detected cancers (SDLC2 and SDLC4). Next, stratified analyses and multivariable modeling (Table 4) were performed since subtle differences in patient characteristics between the lung cancer case groups could influence outcomes. The results were consistent when we restricted to stage I and II patients (Fig 3A and 3B) and when we stratified by histology (S3 and S4 Figs) despite finding a higher percentage of squamous cell carcinomas for SDLC3/SDLC4 and a higher percentage of adenocarcinomas for SDLC1/SDLC2. Thus, stage and histology were not contributing to the observed survival differences. Finally, the results were consistent when multivariable models (Table 4) were utilized to remove the influence of potential prognostic factors. Thus, our systematic analyses appeared to rule out sources of biases and confounding that could have attributed the observation that patients with at least one antecedent negative screen exhibited significantly poorer survival when compared to patients with consecutive positive screens prior to cancer diagnosis.

As defined by the NLST [1, 3], screen-detected cancers were defined by the sequence of screen positivity and negativity. A newly positive screen could represent either a new finding satisfying definition of screen positivity (a nodule 4 mm or larger or a finding suspicious for lung cancer) or the evolution of a nodule previously not fulfilling definition of positive screen (nodule < 4 mm) that evolved. In rare instances, a clearly abnormal finding on a screen could have been missed by the readers—a false negative interpretation. Anatomic location did not play a role in determining screen positivity. As such, we acknowledge the limitation that a nodule prompting a positive screen on an antecedent screen could be in a different anatomic location than the nodule prompting a positive screen at a later time point. The SDLC1/SDLC2 cases had positive screens at baseline and depending upon the features of the detected nodules prompting a positive screen, these participants would have undergone additional evaluation, typically imaging-based, but occasionally histologic sampling at T0 (< 11%). Among the 104 lung cancer patients in SDLC1, 103 had a biopsy at T1 and only 6 had a biopsy at T0; 88 of 92 patients in SDLC2 had a biopsy at T2, 6 had a had a biopsy at T1, and only 3 had a biopsy at T0. In the absence of evolutionary changes in nodule features on imaging (increased size or attenuation) or confirmatory histology, positive screens would not undergo more aggressive management, but would receive next annual screen at T1. The SDLC3/4 cancers followed negative baseline screens which is also based on NLST criteria [1] where either “no or minor abnormalities” that fell below the threshold criteria for screen positivity (S1 Table). Although the numbers of screen-detected lung cancers arising in the setting of one or more prior negative screens is small, our data supports a more aggressive cancer behavior relative among these screen-detected cancers.

A previous study by Carter *et al*. [4] found that screen-detected lung cancers detected were smaller and more invasive than those diagnosed at prevalence screens. Our analyses revealed that the prevalence lung cancers were larger than the SDLC1/SDLC2 and SDLC3/SDLC4 lung cancers (Table 3), had a higher percentage of adenocarcinomas compared to SDLC3/SDLC4, and exhibited poorer outcomes than the SDLC1/SDLC2 lung cancer but improved outcomes compared to SDLC3/SDLC4 cancers. Our analyses also revealed that interval cancers were associated with worst outcome which is likely attributed to that these cancers exhibited a shift towards more biologically aggressive SCLC histology, had significantly larger tumors, and had advanced stage disease at time of diagnosis.

There are limitations of our analysis. The generalizability of our results to screening populations outside the NLST eligibility criteria is indeterminate [3]. Although we performed stratified and multivariable analyses, we cannot account for biases from unknown confounders and unmeasured covariates. For instance, it is unknown to what degree over-diagnoses occur in the SDLC1/2 cases, or whether they would have remained biologically indolent had they not been treated. As noted, we do not know whether the nodules sequentially observed in SDLC1/2 lung cancer patients were the same nodules that progressed and developed into lung cancer. Moreover, the implications of changing threshold criteria for screen positivity on outcomes have yet to be determined prospectively. However, our results reaffirm that interval cancers are more aggressive, and that screen-detected cancers in cohorts with antecedent negative screens show histologic differences relative to cancers observed at prevalence or screen-detected screens with consistently prior positive screens, appear to be more aggressive, and to suffer survival disadvantages.

Although the NLST demonstrated a clear benefit of lung cancer and all-cause mortality reduction with CT screening [1], this *post hoc* analysis reveals important and novel insight to the heterogeneity of lung cancers diagnosed in a screening population. As with interval cancers diagnosed following a negative screen, *de novo* lung tumors that arise in a lung environment ostensibly free of lung nodules are likely more rapidly growing and aggressive which results in the significantly poorer outcomes. These findings could support more aggressive treatment of interval cancers and *de novo* screen-detected cancers with antecedent negative screens, since current evidence indicates that adjuvant chemotherapy confers a survival advantage for NSCLC patients with high-risk disease [16, 17]. However, a recent *post hoc* analysis by Patz et al [12] suggested that annual screening after a negative screen might be unnecessary since the authors reported a significant reduction in lung cancer incidence among NLST participants with a baseline negative screen compared to those with a baseline positive screen. Ideally though, molecular, genetic, and imaging-based biomarkers should be developed to assist in identifying high-risk participants and biologically aggressive nodules [18, 19]. As such, additional research will be needed inform the potential translational implications of these findings, to understand the biology of these screen-detected tumors, to determine whether these findings are consistent across screening trials and screening thresholds, and how to personalize cancer management in these potentially vulnerable patients.

## Supporting Information

S1 Fig Schema for the Entire CT-arm of the NLST based on Screening Results and Lung Cancer Diagnoses. The dashed lines indicate the parts of the schema that were not included in the final analyses.
(PDF)

S2 Fig (A) Kaplan-Meier Estimates of Progression Free Survival with Number of Subjects at Risk for the Individual Screen-Detected Cancer Cohorts and Prevalence Cancer Cohort. (B) Kaplan-Meier Estimates of Overall Survival with Number of Subjects at Risk for the Individual Screen-Detected Cancer Cohorts and Prevalence Cancer Cohort. (DOCX)

S3 Fig (A) Kaplan-Meier Estimates of Progression Free Survival of Adenocarcinoma/BAC Cases With Number of Subjects at Risk within the Prevalence and Combined Screen-Detected Cancer Cohorts. (B) Estimates of Progression Free Survival of Adenocarcinoma/BAC Cases With Number of Subjects at Risk within the Prevalence and Combined Screen-Detected Cancer Cohorts. (DOCX)

S4 Fig (A) Kaplan-Meier Estimates of Progression Free Survival for Squamous Cell Carcinoma Cases With Number of Subjects at Risk within the Prevalence and Combined Screen-Detected Cancer Cohorts. (B) Kaplan-Meier Estimates of Overall Survival for Squamous Cell Carcinoma Cases with Number of Subjects at Risk within the Prevalence and Combined Screen-Detected Cancer Cohorts. (DOCX)

S1 Table CT Screening Results at Each Round for the Incidence Cancer Cohorts with Lung Cancer. Abbreviations: SDLC1 = screen-detected lung cancers cohort 1 with baseline positive screens not associated with a lung cancer diagnosis and a screen-detected incidence lung cancer followed a positive screen at T1; SDLC2 = screen-detected lung cancers cohort 2 with baseline and T1 positive screens not associated with a lung cancer diagnosis and a screen-detected incidence lung cancer followed a positive screen at T2; SDLC3 = screen-detected lung cancers cohort 3 with baseline negative screens and a screen-detected incidence lung cancer followed a positive screen at T1; SDLC4 = screen-detected lung cancers cohort 4 with baseline and T1 negative screens and a screen-detected incidence lung cancer followed a positive screen at T2; PC = Prevalence lung cancer cohort.
(DOCX)

S2 Table Time Intervals Between Events for the Incidence Lung Cancer Cohorts. (DOCX)
