# Supplementary material for: Differences in Patient Outcomes of Prevalence, Interval, and Screen-Detected Lung Cancers in the CT Arm of the National Lung Screening Trial
Source: PLoS One. 2016 Aug 10;11(8):e0159880. doi: 10.1371/journal.pone.0159880 (PMC4980050; doi:10.1371/journal.pone.0159880)
Supplement: S1 Fig — The dashed lines indicate the parts of the schema that were not included in the final analyses. (PDF) [file pone.0159880.s001.pdf]

**S1 Fig. Schema for the Entire CT-arm of the NLST based on Screening Results and Lung Cancer Diagnoses.** The dashed lines indicate the parts of the schema that were not included in the final analyses

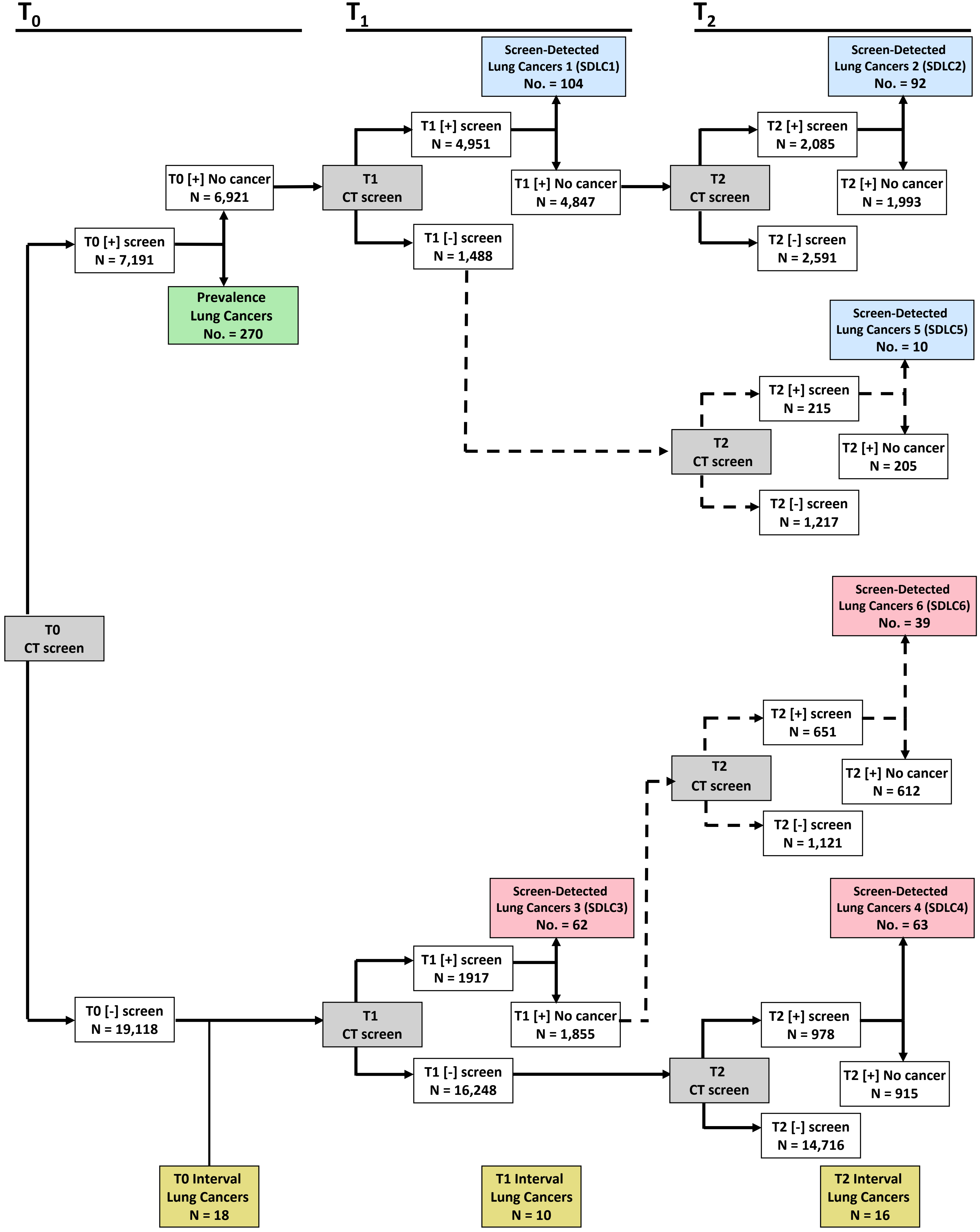

$T_0$
$T_1$
$T_2$
Screen-Detected Lung Cancers 1 (SDLC1) No. = 104
Screen-Detected Lung Cancers 2 (SDLC2) No. = 92
T1 [+] screen N = 4,951
T2 [+] screen N = 2,085
T0 [+] No cancer N = 6,921
T1 CT screen
T1 [+] No cancer N = 4,847
T2 CT screen
T2 [+] No cancer N = 1,993
T0 [+] screen N = 7,191
T1 [-] screen N = 1,488
T2 [-] screen N = 2,591
Prevalence Lung Cancers No. = 270
Screen-Detected Lung Cancers 5 (SDLC5) No. = 10
T2 [+] screen N = 215
T2 CT screen
T2 [+] No cancer N = 205
T2 [-] screen N = 1,217
Screen-Detected Lung Cancers 6 (SDLC6) No. = 39
T0 CT screen
T2 [+] screen N = 651
T2 CT screen
T2 [+] No cancer N = 612
T2 [-] screen N = 1,121
Screen-Detected Lung Cancers 3 (SDLC3) No. = 62
Screen-Detected Lung Cancers 4 (SDLC4) No. = 63
T1 [+] screen N = 1917
T0 [-] screen N = 19,118
T1 CT screen
T1 [+] No cancer N = 1,855
T2 [+] screen N = 978
T2 [+] No cancer N = 915
T1 [-] screen N = 16,248
T2 CT screen
T2 [-] screen N = 14,716
T0 Interval Lung Cancers N = 18
T1 Interval Lung Cancers N = 10
T2 Interval Lung Cancers N = 16
