# Supplementary material for: Differences in Patient Outcomes of Prevalence, Interval, and Screen-Detected Lung Cancers in the CT Arm of the National Lung Screening Trial
Source: PLoS One. 2016 Aug 10;11(8):e0159880. doi: 10.1371/journal.pone.0159880 (PMC4980050; doi:10.1371/journal.pone.0159880)
Supplement: S2 Fig — (DOCX) [file pone.0159880.s002.docx]

**S2 Fig. (A) Kaplan-Meier Estimates of Progression Free Survival with Number of Subjects at Risk for the Individual Incidence Cancer Cohorts and Prevalence Cancer Cohort. (B) Kaplan-Meier Estimates of Overall Survival with Number of Subjects at Risk for the Individual Incidence Cancer Cohorts and Prevalence Cancer Cohort.**

**S2A Fig.**

**S2B Fig.**
