# Supplementary material for: Differences in Patient Outcomes of Prevalence, Interval, and Screen-Detected Lung Cancers in the CT Arm of the National Lung Screening Trial
Source: PLoS One. 2016 Aug 10;11(8):e0159880. doi: 10.1371/journal.pone.0159880 (PMC4980050; doi:10.1371/journal.pone.0159880)
Supplement: S3 Fig — (DOCX) [file pone.0159880.s003.docx]

**S3 Fig. (A) Kaplan-Meier Estimates of Progression Free Survival of Adenocarcinoma Cases With Number of Subjects at Risk within the Prevalence and Combined Incidence Cancer Cohorts. (B) Kaplan-Meier Estimates of Overall Survival for Adenocarcinoma Cases With Number of Subjects at Risk within the Prevalence and Combined Incidence Cancer Cohorts.**

**S3A Fig.**

**S3B Fig.**
