# Supplementary material for: Differences in Patient Outcomes of Prevalence, Interval, and Screen-Detected Lung Cancers in the CT Arm of the National Lung Screening Trial
Source: PLoS One. 2016 Aug 10;11(8):e0159880. doi: 10.1371/journal.pone.0159880 (PMC4980050; doi:10.1371/journal.pone.0159880)
Supplement: S1 Table — Abbreviations: SDLC1 = screen-detected lung cancers cohort 1 with baseline positive screens not associated with a lung cancer diagnosis and a screen-detected incidence lung cancer followed a positive screen at T1; SDLC2 = screen-detected lung cancers cohort 2 with baseline and T1 positive screens not associated with a lung cancer diagnosis and a screen-detected incidence lung cancer followed a positive screen at T2; SDLC3 = screen-detected lung cancers cohort 3 with baseline negative screens and a screen-detected incidence lung cancer followed a positive screen at T1; SDLC4 = screen-detected lung cancers cohort 4 with baseline and T1 negative screens and a screen-detected incidence lung cancer followed a positive screen at T2; PC = Prevalence lung cancer cohort. (DOCX) [file pone.0159880.s005.docx]

| **S1 Table. CT Screening Results at Each Round for the Incidence Cancer Cohorts with Lung Cancer** | | | | | | |
| --- | --- | --- | --- | --- | --- | --- |
| **Screening Result** | | **T0 round** | | **T1 Round** | | **T2 Round** |
|  |  | **SDLC1, N (%)** | **SDLC2, N (%)** | **SDLC1, N (%)** | **SDLC2, N (%)** | **SDLC2, N (%)** |
| **Negative** | No or minor abnormalities not suspicious for lung cancer, or significant abnormalities unrelated to lung cancer | 0 (0.0%) | 0 (0.0%) | 0 (0.0%) | 0 (0.0%) | 0 (0.0%) |
| **Positive** | Nodule(s) ≥ 4 mm, masses, or other abnormalities suspicious for lung cancer | 104 (100.0%) | 92 (100.0%) | 25 (24.0%)^1^ | 11 (12.0%) | 0 (0.0%) |
| **Positive** | Stable abnormalities potentially related to lung cancer; no significant change since prior screening exam | 0 (0.0%) | 0 (0.0%) | 10 (9.6%)^1^ | 37 (40.2%) | 12 (13.0%) |
| **Positive** | New or evolving lesions relative to prior screen, suspicious for lung cancer | 0 (0.0%) | 0 (0.0%) | 69 (66.4%) | 44 (47.8%) | 80 (87.0%) |
|  |  |  | |  | |  |
|  |  | **T0 round** | | **T1 Round** | | **T2 Round** |
|  |  | **SDLC3, N (%)** | **SDLC4, N (%)** | **SDLC3, N (%)** | **SDLC4, N (%)** | **SDLC4, N (%)** |
| **Negative** | No or minor abnormalities not suspicious for lung cancer, or significant abnormalities unrelated to lung cancer | 62 (100%) | 63 (100.0%) | 0 (0.0%) | 63 (100.0%) | 0 (0.0%) |
| **Positive** | Nodule(s) ≥ 4 mm, masses, or other abnormalities suspicious for lung cancer | 0 (0.0%) | 0 (0.0%) | 8 (12.9%) | 0 (0.0%) | 0 (0.0%) |
| **Positive** | Stable abnormalities potentially related to lung cancer; no significant change since prior screening exam | 0 (0.0%) | 0 (0.0%) | 1 (1.6%)^2^ | 0 (0.0%) | 1 (1.6%)^2^ |
| **Positive** | New or evolving lesions relative to prior screen, suspicious for lung cancer | 0 (0.0%) | 0 (0.0%) | 53 (85.5%)^2^ | 0 (0.0%) | 62 (98.4%) |
| ^1^ At the T1 screen, the designation of “Positive, stable screen” was incompletely implemented across all sites; as such, some T1 screens with stable findings were merely reported as “Positive, nodule(s) ≥ 4 mm, etc.”.  ^2^  In these cohorts, prior screens were reported as negative, but may have contained nodules < 4 mm or missed nodules that were reported to be stable or evolving on the next annual screen. | | | | | | |
