# Supplementary material for: Differences in Patient Outcomes of Prevalence, Interval, and Screen-Detected Lung Cancers in the CT Arm of the National Lung Screening Trial
Source: PLoS One. 2016 Aug 10;11(8):e0159880. doi: 10.1371/journal.pone.0159880 (PMC4980050; doi:10.1371/journal.pone.0159880)
Supplement: S2 Table — (DOCX) [file pone.0159880.s006.docx]

| **S2 Table. Time Intervals Between Events for Each Incidence Lung Cancer Cohort** | | | | | |  |
| --- | --- | --- | --- | --- | --- | --- |
|  |  | |  |  | | |
|  | **T1 Incidence Screens** | |  | **T2 Incidence Screens** | | |
|  | | | | | |  |
| Time (months) from randomization to cancer diagnosis for each incidence lung cancer case cohort | | | | | |  |
| **Variable** | **SDLC1** | **SDLC3** |  | **SDLC2** | **SDLC4** | |
| Mean (SD) | 13.0 (1.82) | 13.0 (2.02) |  | 25.3 (2.77) | 24.9 (1.58) | |
| P-value^1^ | 0.83 | |  | 0.74 | | |
|  |  |  |  |  |  | |
| Median (95% CI) | 12.5 (12.19 - 12.91) | 12.4 (12.05 - 12.88) |  | 24.5 (24.15 - 24.74) | 24.5 (24.05 - 24.84) | |
| P-value^2^ | 0.66 | |  | 0.69 | | |
|  | | | | | |  |
|  | | | | | |  |
| Time interval (months) between two successive screens | | | | | |  |
|  | **T_0_ to T_1_ for SDLC1** | **T_0_  to T_1_ for SDLC3** |  | **T_1_  to T_2_ for SDLC2** | **T_1_  to T_2_ SDLC4** | |
| Median (IQR) | 12.2 (11.3-12.9) | 12.0 (11.3-12.9) |  | 12.1 (11.3-13.1) | 12.2 (11.6-12.6) | |
| P-value^2^ | 0.66 | |  | 0.95 | | |
|  |  |  |  |  |  | |
| Months, N (%) |  |  |  |  |  | |
| < 3 months | 0 (0.0) | 0 (0.0) |  | 0 (0.0) | 0 (0.0) | |
| ≥ 3 to < 6 months | 0 (0.0) | 0 (0.0) |  | 0 (0.0) | 0 (0.0) | |
| ≥ 6 months | 104 (100%) | 62 (100%) |  | 92 (100%) | 63 (100%) | |
| **Abbreviations:** SD, standard deviation; CI, confidence interval; IC = incidence cancers; IQR = interquartile range  ^1^ P-value from the Van Der Waerden normal scores test comparing SDLC1 vs. SDLC3 and SDLC2 vs. SDLC4  ^2^ P-value from the Wilcoxon signed-rank test  ^3^ P-value from Pearson’s chi-square | | | | | |  |
